# Interference with DNA repair after ionizing radiation by a pyrrole-imidazole polyamide

**DOI:** 10.1371/journal.pone.0196803

**Published:** 2018-05-01

**Authors:** Silvia Diaz-Perez, Nathanael Kane, Alexis A. Kurmis, Fei Yang, Nicolas T. Kummer, Peter B. Dervan, Nicholas G. Nickols

**Affiliations:** 1 Department of Radiation Oncology, University of California, Los Angeles, California, United States of America; 2 Division of Chemistry and Chemical Engineering, California Institute of Technology, Pasadena, California, United States of America; 3 Department of Radiation Oncology, VA Greater Los Angeles Healthcare System, Los Angeles, California, United States of America; University of South Alabama Mitchell Cancer Institute, UNITED STATES

## Abstract

Pyrrole-imidazole (Py–Im) polyamides are synthetic non-genotoxic minor groove-binding small molecules. We hypothesized that Py–Im polyamides can modulate the cellular response to ionizing radiation. Pre-treatment of cells with a Py-Im polyamide prior to exposure to ionizing radiation resulted in a delay in resolution of phosphorylated γ-H2AX foci, increase in XRCC1 foci, and reduced cellular replication potential. RNA-sequencing of cell lines exposed to the polyamide showed induction of genes related to the ultraviolet radiation response. We observed that the polyamide is almost 10-fold more toxic to a cell line deficient in DNA ligase 3 as compared to the parental cell line. Alkaline single cell gel electrophoresis reveals that the polyamide induces genomic fragmentation in the ligase 3 deficient cell line but not the corresponding parental line. The polyamide interferes directly with DNA ligation *in vitro*. We conclude that Py-Im polyamides may be further explored as sensitizers to genotoxic therapies.

## Introduction

Half of all cancer patients undergo radiotherapy [1]. A primary mechanism of action of radiotherapy is induction of DNA damage. Combinations of radiation with radiosensitizing drugs is a fundamental treatment paradigm in clinical oncology [2]. Most systemic therapies currently used as radiosensitizers (e.g., flurouracil, cisplatin, mitomycin, gemcitabine, topoisomerase poisons) interfere with DNA repair as part of their mechanism of radiosensitization [3]. However, all of these drugs are genotoxic. Non-genotoxic small molecules that potentiate the effects of ionizing radiation in malignant cells could be used to augment radiotherapy for solid tumors.

Pyrrole-imidazole (Py–Im) polyamides comprise a class of small molecule minor groove-binders that are non-genotoxic [4]. Py-Im polyamides, oligomers of aromatic amino acids linked in series, fold into an antiparallel hairpin structure upon binding DNA [4]. The side-by-side pairings of the Py and Im subunits determine DNA target sequence specificity. The ring-pairs conform to steric and hydrogen bonding pattern differences in the floor of the minor groove presented by the edges of the base pairs [5]. DNAse I footprinting titrations and other methods have established the binding affinity and specificity of these molecules [6]. Protein-DNA interactions can be inhibited by allosteric changes in the DNA minor and major grooves induced by Py-Im polyamide-DNA binding [7]. Py-Im polyamides composed of eight aromatic rings localize to the nucleus in live cells [8]. Py-Im polyamides fail to activate a canonical DNA damage response [9], are not genotoxic on their own [10,11], and do not significantly alter cell cycle distribution at concentrations used for gene expression studies [9]. Py-Im polyamides have been used as molecular probes in cell culture to modulate gene-expression pathways [12,13] and interfere with RNA Polymerase II elongation [10,14].

We hypothesized that a Py–Im polyamide could modulate the cellular response to ionizing radiation. Pre-treatment of LNCaP and VCaP cells with a Py-Im polyamide prior to exposure to ionizing radiation resulted in a delay in resolution of phosphorylated γ-H2AX foci indicative of delayed repair of double strand breaks, and increased induction of XRCC1 foci consistent with a higher frequency of single strand breaks. RNA-sequencing of cell lines treated with the polyamide showed induction of genes related to the ultraviolet radiation response. We observed the polyamide is almost 10-fold more toxic to a LN-428 cell line deficient in DNA ligase 3 as compared to its parental cell line. Alkaline comet assay reveals that the polyamide induces genomic fragmentation in the Ligase 3 deficient but not the parental line. The polyamide interferes directly with DNA ligation *in vitro*. We conclude that Py-Im polyamides may be further explored as sensitizers to genotoxic therapies.

## Materials and methods

### Cell culture

Early passage LNCaP (ATCC, CRL-1740) and VCaP (ATTCC CRL-2876) were cultured in RPMI 1640 with 10% FBS and DMEM with 10% FBS, respectively. LN428 glioma cell lines KD-BER-LN428-control and KD-BER-LN428-LIG3 (Trevigen 54999-001-01 and 5504-001-01) were cultured in alpha MEM medium supplemented with 10% Heat Inactivated FBS, 10 mg/ml Gentamycin, 1 μg/ml Puromycin.

### Immunofluorescence

Irradiation was performed using a laboratory irradiator (Gulmay Medical). Immunofluorescence was performed on cells grown on cover slips coated with 0.1 mg/ml of Poly-D-lysine, fixed with 4% paraformaldehyde (electron microscopy science) in PBS for 15 min at room temperature. Cell permeabilization was performed with 0.5% ice-cold Triton X-100 for 15 min at room temperature. Cells were incubated with blocking solution (10% FBS, heat inactivated on 0.05% Tween on PBS) for 1 hour at room temperature. Cover slip were incubated with 1:30 dilution with primary antibodies anti γ-H2AX-FITC (Millipore, # 16-202A), Anti-XRCC1 (Novus, # NB100-532), 4°C overnight. Anti-XRCC1 was detected with 1:200 dilution of donkey anti-Rabbit DyLight 594 (Novus, # NB1P1-75642). Nuclei were counterstained with DAPI, viewed with Leica DMR fluorescent microscope, images captured with Quips mFISH software (Vysis). Three fields were selected at random and 10 nuclei per field were counted.

### Cell viability measured using xCELLigence

The xCELLigence system noninvasively monitors viability of cultured cells by impedance, quantified as cell index (CI), representing cell number, viability, morphology. Assays were in 96 well plates with KD-BER-LN428-control, KD-BER-LN-428-LIG3 cells at 6000 cells/well. Varying concentrations of **1** (0.3. 0.1, 0.3, 1.0, 3.0 10, 30 μM) were added, incubated at 37°C with readout every 5 minutes for 72 hours. The experiment was run in biological triplicate.

### Cell viability assay with PrestoBlue

Exponentially growing LNCaP cells on 6 well plates were treated with 10 μM **1** for 24 hours and then irradiated. The cells were pelleted and resuspended twice to remove polyamide from the media, plated at 4000 and 8000 cells/mL in 96 well plates. The plates were incubated at 37°C for 2 weeks, PrestoBlue (Invitrogen) was added and incubated 30 min at room temperature, fluorescence read at 560 nm by spectrophotometry (SPECTRMax).

### Alkaline comet assay

Alkaline comet assay was perform as described [15] using a Trevigen kit (4250-050-K). After treatment with **1** or vehicle, cells were centrifuged. Cells suspension of 1x10^5^/ml in PBS (Ca^++^and Mg^++^ free) were embedded in 300 μl of 1% low-melt agarose at 37°C and 50 μL were mounted on CometSlides (Trevigen) pre-incubated at 37°C. Embedded cells were lysed at 4°C for 60 minutes in the dark, treated 20 min in alkaline unwinding solution (200mM NaOH, 1 nM EDTA pH>13.3) at room temperature in the dark, and electrophoresed (21V for 30 minutes) in a pre-chilled apparatus with fresh un-winding buffer as previously described. Slides were fixed in 70% ethanol for 5 minutes, dried at 37°C, stained with 100 l of 2X diluted SYBR Gold (Invitrogen) for 30 minutes. Slides were imaged with a Leica DMR fluorescent microscope and quantified using OpenComet in ImageJ.

### DNA T4 Ligase experiments

Sequences of oligonucleotides used in ligation assay are: 1a FAM, FAM-GACGCAAGTTCAGCTCGA; 1b CAAGTTCAGACGC; 2a CTGCGTTCAAGTCGAGCTGTTCAAGTCTGCG (Integrated DNA Technology). Ligation was performed in presence of varying concentrations of **1** and **2**, 100 nM of the annealing oligos and 4U of T4 ligase for 1 hour at room temperature. The ligation was stop by adding 5 μl of TBE-Urea sample buffer and incubation at 70°C for 3 minutes. The ligation products were analyzed by acrylamide electrophoresis on a 15% TBE-Urea gel [16] at 180 V for 1 hour. Image acquisitions of the gels were done by Typhoon Imaging System and image quantification by Quant Software.

### RNA sequencing and analysis

LNCaP and VCaP cells were plated at 5 × 10^4^ cells/mL in 10-cm^2^ dishes, treated with or without 10 μM **1** in RPMI 1640 and DMEM supplemented with 10% FBS, respectively, for 24 hours. Total RNA was TRIzol extracted, sequenced (Illumina HiSeq2000), and mapped against the human genome (hg19) with Tophat2 using Ensembl GRCh37 gene annotations. Htseq-count was used for exon alignment and DESeq2 for differential expression. Pathway analysis was performed with the gene set enrichment analysis (GSEA) software on genes with padj<0.05 and p<0.05 for LNCaP and VCaP, respectively.

### Cell cycle

Cells cultured at 70% confluence were treated with 10 μM **1** for 48 hours and cell cycle distribution assessed by monoparametric propidium iodide flow cytometry, analyzed by FacScan I (Becton Dickinson) and ModFit software.

## Results

### Polyamide 1 slows resolution of histone γ-H2AX foci after irradiation

**Fig 1:**
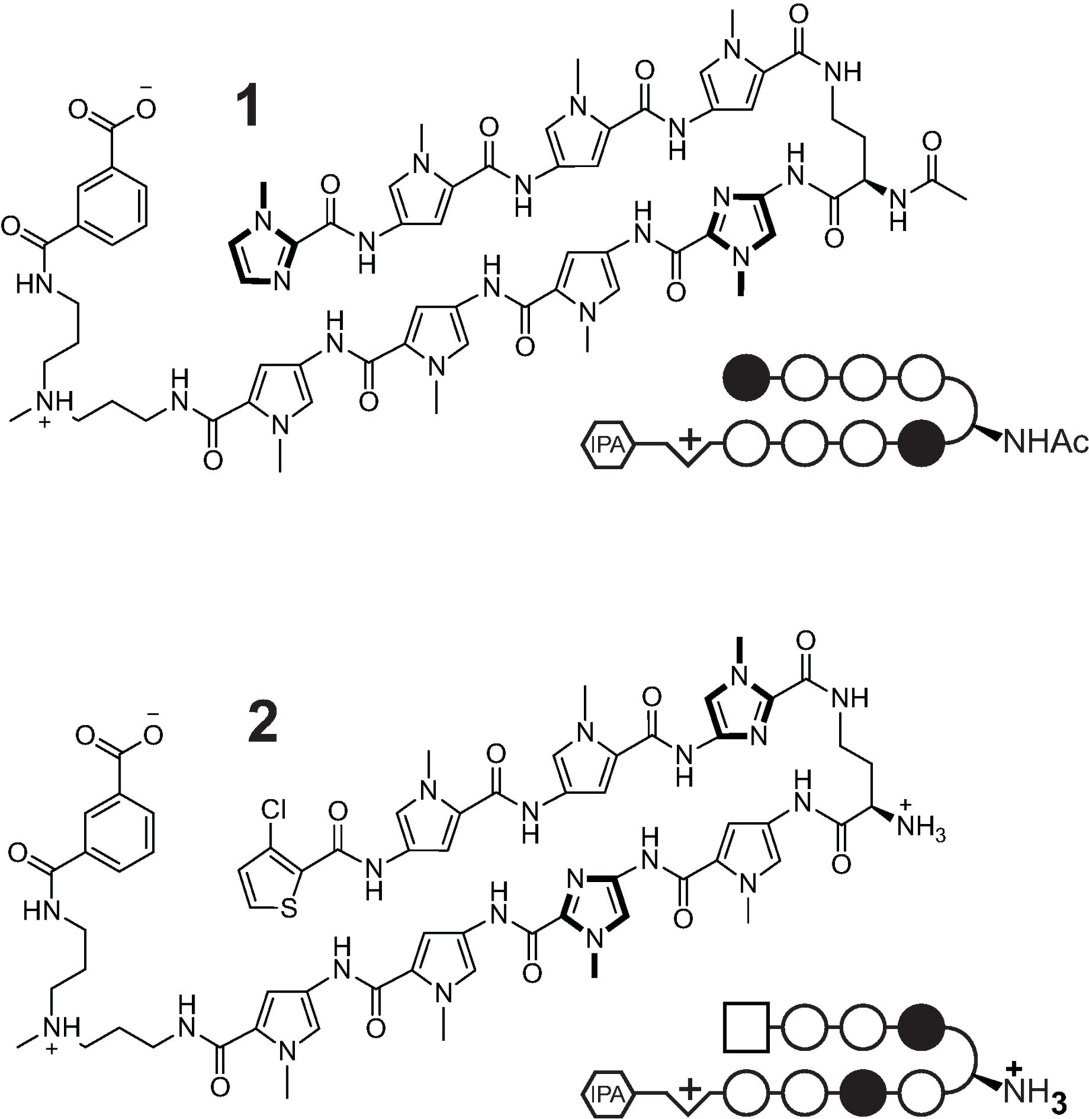
Chemical structures and ball-and-stick models of polyamides 1 and 2. Open circles, closed circles, and square represent pyrrole, imidazole, and chlorothiophene monomers, respectively.

**Fig 2:**
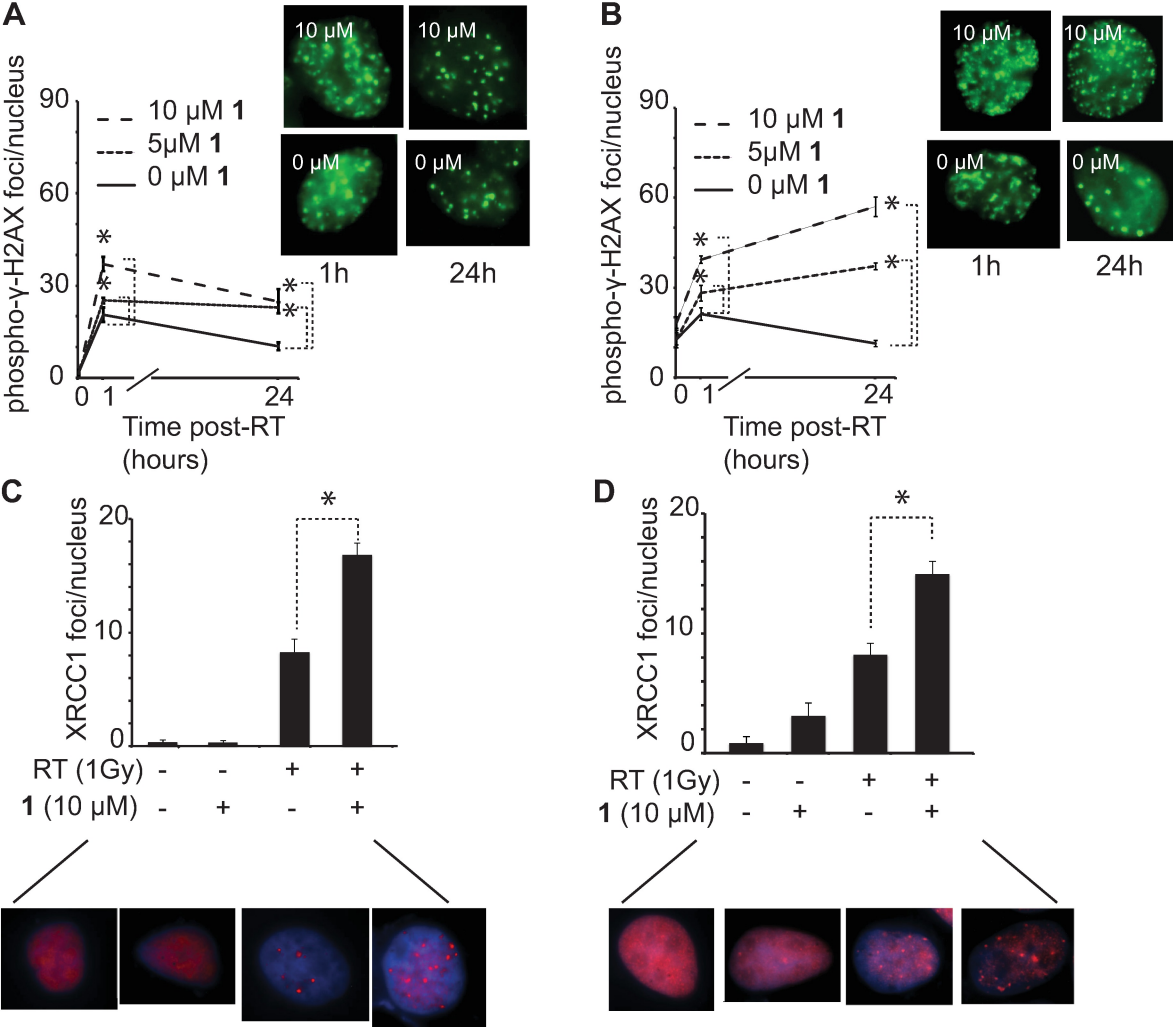
Double and single strand break foci after irradiation. Pretreatment of LNCaP cells (A) and VCaP cells (B) with **1** followed by irradiation (10 Gy) and immunostaining to quantify foci of phosphorylated γ-H2AX. Increased foci indicate unrepaired double strand breaks in cells pretreated with **1** followed by irradiation. XRCC1 foci representative of foci of single strand break repair are also increased by pretreatment with **1** followed by irradiation (1 Gy) in LNCaP cells **(C**) and VCaP cells (D). Three fields were selected at random and at least 10 nuclei per field were counted. * p < 0.0001. Error bars are 95% CI. Representative cells before irradiation are included in the supporting information.

Polyamide **1** (Fig 1) does not cause genomic fragmentation by alkaline comet assay [11]. However, we hypothesized that **1** may interfere with repair of DNA after genotoxic insult. We examined the effect of **1** on the DNA double strand break repair dynamics in LNCaP and VCaP lines exposed to ionizing radiation. Phosphorylated γ-H2AX was used as a marker for double strand breaks [17]. LNCaP and VCaP cell lines grown with 5 and 10 μM **1** for 24 hours were irradiated (10 Gy) and immune-stained at baseline and after 1 and 24 hours with anti-γ-H2AX antibody. Phosphorylated γ-H2AX foci increased dramatically from baseline and remained elevated at 24 hours. Cells pre-treated with **1** had higher levels of phosphorylated γ-H2AX at both time points (Fig 2A and 2B). LNCaP cells subsequently exhibited reduced long-term proliferation after irradiation if pre-treated with **1** (S1 Fig). Because the radiosensitivity can be cell cycle dependent [18], we investigated the impact of **1** on cell cycle distribution in LNCaP and VCaP cells. We observed minimal change in cell cycle distribution after treatment with **1** for 48 hours at 10 μM (S2 Fig), consistent with prior reports of a related polyamide at this concentration and time-course [9].

### Polyamide 1 increases foci of single strand break repair rapidly after irradiation

Ionizing radiation induces at least an order of magnitude greater number of DNA single strand breaks than double strand breaks [19], although the latter are thought to be the lethal lesion, as single strand breaks are rapidly repaired [20]. XRCC1 was used as a marker for single strand breaks. XRCC1 is recruited to sites undergoing single–strand break repair by poly(ADP-ribose) polymerase 1 (PARP-1), responsible for the initial recognition of the break [21,22]. Once XRCC1 is bound to the single strand break, it serves as a scaffolding platform to recruit, activate, regulate downstream repair enzymes. In order to assess the effect of **1** on the formation of single strand breaks after ionizing radiation, we measured the nuclear recruitment of XRCC1 (Fig 2C and 2D). LNCaP and VCaP were grown with 5 and 10 μM **1** for 24 hours, irradiated (1 Gy), and immuno-stained after 4 min with anti-XRCC1 antibody. Treatment of LNCaP with **1** alone resulted in no increase in XRCC1 foci, while VCaP cells had a small increase over baseline. Pretreatment of both cell lines with **1** prior to irradiation resulted in a large increase in XRCC1 foci as compared to irradiation alone.

### siRNA knockdown of DNA ligase 3 potentiates genotoxicity by Py-Im polyamide 1

**Fig 3:**
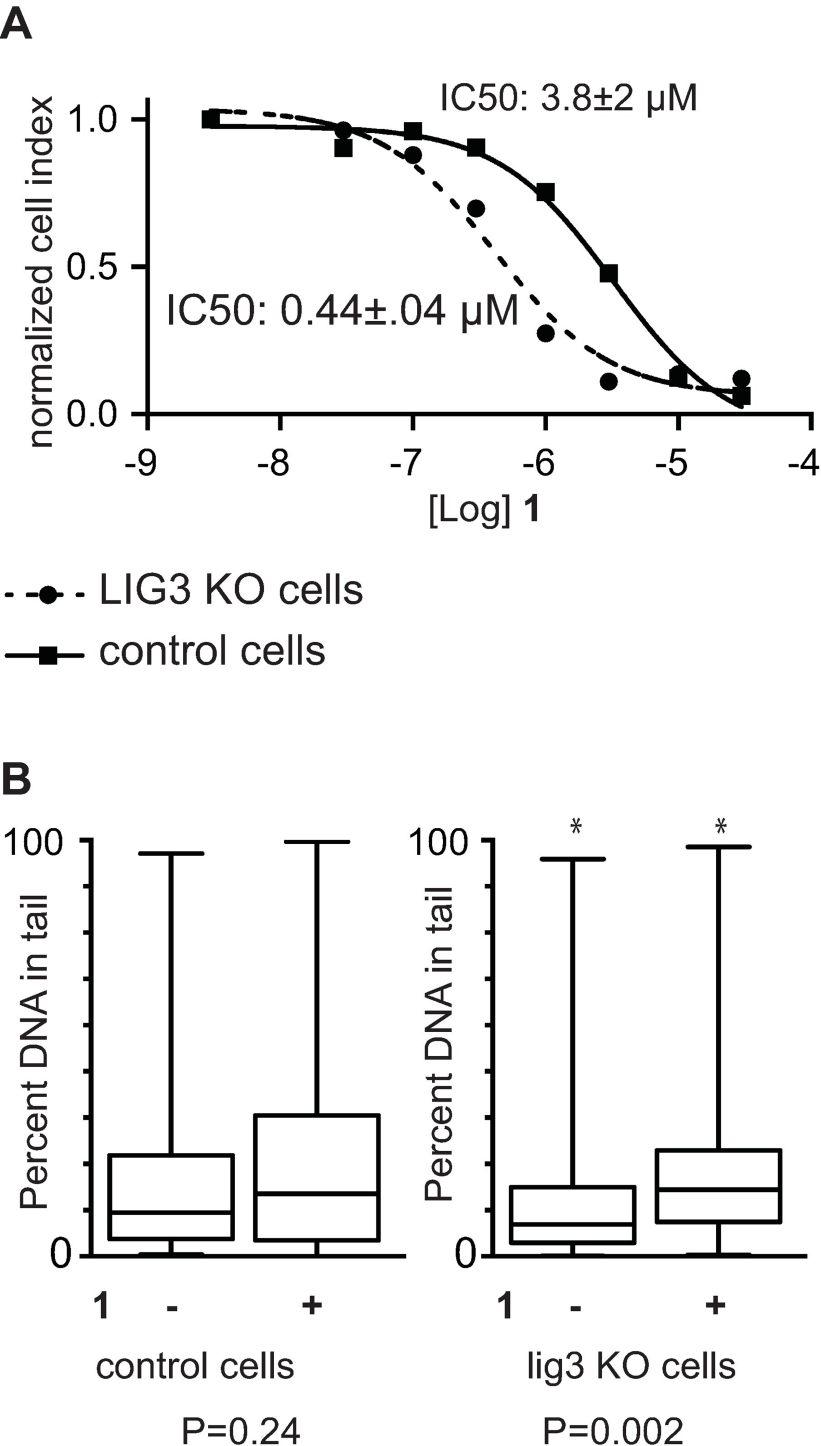
The effect of DNA Lig3 knockdown on cytotoxicity and genotoxicity by polyamide 1. KD-BER-LN-428-LIG3 cells stably express siRNA against DNA lig3 and KD-BER-LN-428-control cells do not. Cells were grown in the presence of **1** at varying concentrations for 72 hours. (A) The IC50s of **1** are 3.8±2 and 0.44±0.4 μM in control and lig3 deficient cells, respectively. Errors represent 95% CI. (B) Alkaline comet assay of KD-BER-LN-428-LIG3 and KD-BER-LN-428-control cells after treatment with **1** for 24 hours. **1** increases genomic fragmentation in the LIG3 deficient but not control cells. Whisker plots show upper and lower bounds, quartiles, means. At least 180 comets were evaluated per condition. Representative comets are included in supporting information.

DNA ligase 3 (LIG3) is involved in DNA replication and repair including of both single and double strand breaks [23]. We investigated if deficiency in LIG3 could potentiate the cytotoxicity of **1** using the KD-BER-LN-428-control (LN428-control) and KD-BER-LN-428-LIG3 (LN428-LIG3) cell line pair. LN-428-LIG3 stably expresses siRNA against *lig3* resulting in >80% knockdown of *lig3* transcript and protein expression as compared to the parental cell line (KD-BER-LN428-control). Cytotoxicity of **1** was measured in both lines using the xCELLigence system. LN428-LIG3 was 8.6-times more sensitive (Fig 3A) to **1** compared to LN428-control. To determine if reduced levels of LIG3 are associated with increased genotoxicity upon treatment with **1**, both cell lines were treated with 10 μM **1** or vehicle for 24 hours and assessed by alkaline comet assay. We observed that **1** increases genomic fragmentation in LN-428-LIG3 but not LN428-control (Fig 3B).

### Sequence dependent interference with DNA ligation *in vitro*

**Fig 4:**
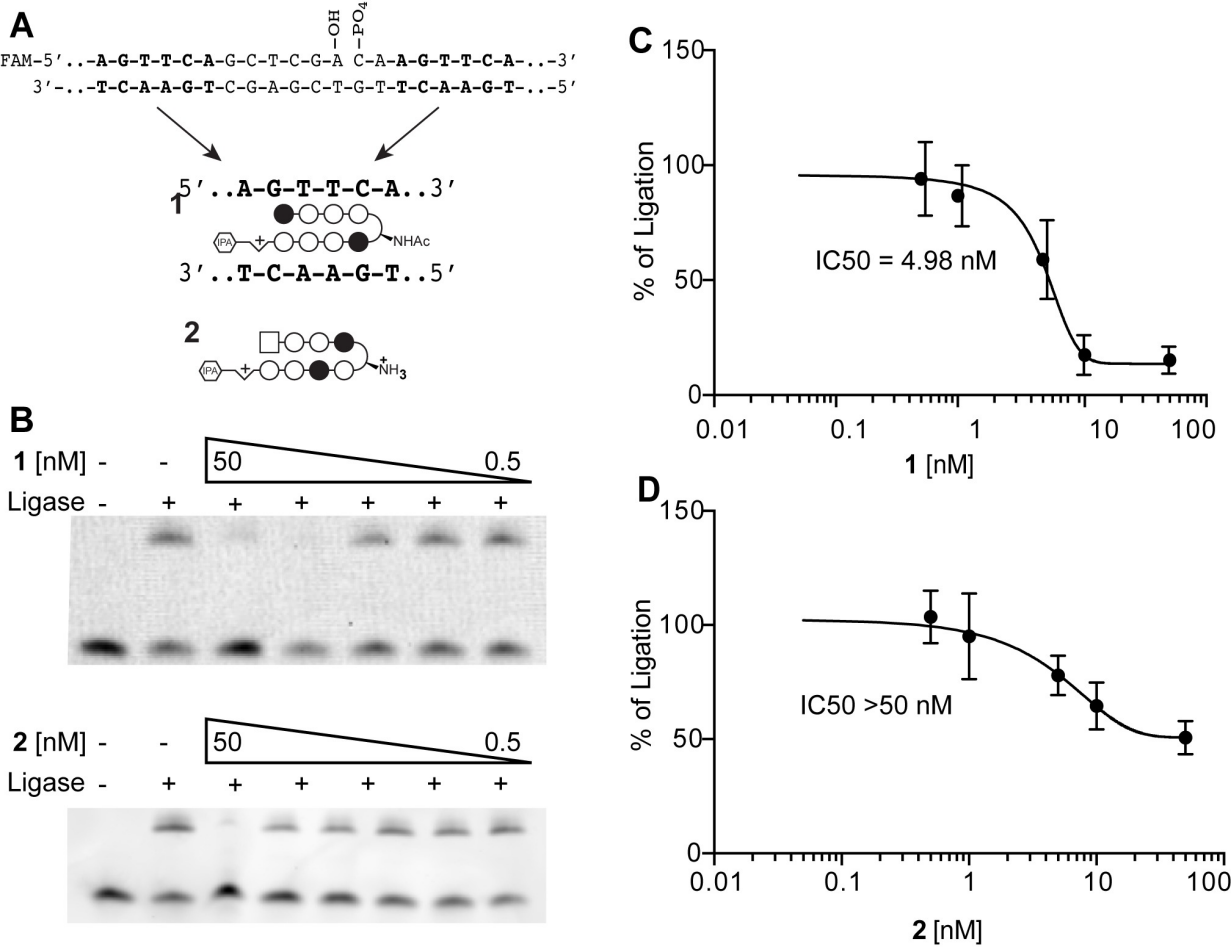
Polyamide 1 inhibits DNA ligation. (A) 5’ FAM labeled oligo duplex containing two binding sites for **1,** but no sites for **2**, adjacent to a single strand nick (top strand). (B) Interference with DNA ligation by **1** and **2** was assessed by gel electrophoresis. (C) Polyamide **1** inhibited the ligation with an IC50 of 4.89 nM; **2**, which targets a sequence not in the duplex, has an IC50 >50 nM. Gels are representative of three independent experiments.

The ATP-dependent DNA ligases catalyze the joining of single-stranded breaks (nicks) in the phosphodiester back-bone of double-stranded DNA. We investigated if **1** can interfere with the DNA ligation *in vitro* using a fluorescent labeled 30-mer oligonucleotide with a nick in a top strand adjacent to flanking binding sites for **1**. Ligation by T4 Ligase was inhibited by polyamide **1** but not **2**, a polyamide which targets an unrelated sequence (Fig 4).

### Polyamide 1 induces a transcriptional response in cells associated with the UV response

We performed gene expression profile analysis in both LNCaP and VCaP cells treated with **1** for 24 hours [24]. GSEA of affected genes for pathways in the Molecular Signatures Database revealed, for both cell lines, DAcosta_UV_response_via_ercc3_dn was the most negatively enriched, and Dacosta_UV_response_via_ercc3_up was most enriched in VCaP (S3 Fig). *Ercc3* codes for xeroderma pigmentosum type B, which is involved in basal transcription and single strand break repair. UV irradiation generates a number of DNA damage lesions including cyclobutane-pyrimidine dimers and 6–4 photoproducts [25]. These lesions are repaired through single strand break repair pathways that require the activity of DNA ligases [25]. The downstream transcriptional consequences of interference with these repair pathways by 1 may resemble those induced by UV irradiation. Similar GSEA results for polyamides have been reported in other cell and tumor samples [24].

## Discussion

We have previously shown that polyamides can modulate a variety of DNA-dependent processes, including transcription factor-DNA binding [12,13], RNA polymerase II elongation [10,14], DNA helicase activity [9], and integration of viral DNA into mammalian cells by integrase [26]. We now report *in vitro* data that a polyamide can interfere with DNA ligation when bound adjacent to sites of single strand breaks, *in vivo* data that a polyamide can inhibit DNA repair following genotoxic insult, and that siRNA knockdown of DNA ligase 3 increases polyamide cytotoxicity by >8-fold, associated with evidence of genomic fragmentation.

Similar to prior reports [11], we find that the polyamide is not inherently genotoxic in cells with intact DNA repair mechanisms. However, the polyamide can potentiate the genotoxicity of ionizing radiation. Polyamide treatment of cells followed by exposure to ionizing radiation immediately increases foci of XRCC1 representative of single strand breaks and increases persistent foci of phosphorylated γ-H2AX representative of unrepaired double strand breaks. Overall, the data suggest a model where a polyamide may interfere with repair of single strand breaks induced after ionizing radiation. Unrepaired single strand breaks in close proximity may become double strand breaks, which can cause cell death and reduced replication potential.

A prior study from our group showed that a related polyamide could exert low-level replication stress in cells accompanied by activation of ATR but not ATM, and at high concentrations (30–100 μM), led to accumulation of cells in S phase without detectable genotoxicity [9]. *In vitro* data showed that the polyamide could slow DNA unwinding by T7 gp4A helicase. Direct interference with DNA ligation of Okazaki fragments on the lagging strand by local polyamide binding offers a complementary explanation for these observations, and is consistent with our current results.

Cancers deficient in DNA repair pathways are often selectively sensitive to DNA damaging drugs and inhibitors of DNA repair [27]. Examples of this strategy include the use of PARP inhibitors or platinating agents for patients with DNA repair deficient breast [28], ovarian [29], and prostate cancers [30,31]. In LN428 cells stably expressing siRNA against DNA Lig3, polyamide treatment results in genomic fragmentation and increased cytotoxicity that is absent when DNA Lig3 is expressed at basal levels. To our knowledge, this is the first observation of a DNA repair deficient cell line with increased sensitivity to a polyamide. Although DNA ligase deficiency is a rare feature of cancers, the observation that reducing a cancer cells ability to enzymatically manipulate its DNA may selectively increase the cytotoxicity of a polyamide raises the question if polyamides may act synergistically with therapeutics that cause replications stress or interfere with DNA repair, in addition to therapies that induce genotoxic stress such as ionizing radiation.

## Supporting information

S1 Fig Cellular proliferation of LNCaP cells after 24 hours pre-treatment with 1 followed by irradiation. After irradiation, cells were washed twice and fresh media replaced without **1**, re-plated at 4000 and 8000 cells/mL in 96 well plates and grown for 14 days. Proliferation was assessed by PrestoBlue assay. * p < 0.01.
(TIF)

S2 Fig Cell cycle effects of polyamide 1. LNCaP and VCaP cells were grown in the presence of polyamide **1** at 5 and 10 μM or vehicle for 48 hours. **1** did not affect the relative distribution of cells in G1, S, or G2 phase in VCaP cells. In LNCaP cells, we observed a small decrease in S phase and small increase in G1, which would not be expected to contribute to increased radiosensitivity.
(TIF)

S3 Fig GSEA analysis of 1 in LNCaP and VCaP cells. Dacosta_UV_response_via_ercc3_up was the gene set most positively enriched in LNCaP with an enrichment score of 4.47. This set was also enriched in VCaP cells (enrichment score of 4.09). DAcosta_UV_response_via_ercc3_dn was the most negatively enriched in both cell lines.
(TIF)

S4 Fig Representative cells pre-irradiation (corresponding to Fig 2A). LNCaP and VCaP cells were grown in the presence of polyamide **1** at 5 and 10 μM or vehicle for 24 hours and evaluated by immunostaining for phosphorylated γ-H2AX. (A) LNCaP vehicle. (B) LNCaP 5 μM **1**. (C) LNCaP 10 μM **1**. (D) VCaP 0 μM **1**. (E) VCaP 5 μM **1**. (F) VcAP 10 μM **1**.
(TIF)

S5 Fig Representative comets (corresponding to Fig 3B). KD-BER-LN-428-LIG3 and KD-BER-LN-428-control cells after treatment with **1** for 24 hours were evaluated by comet assay as described in the manuscript. (A) KD-BER-LN-428-control cells with vehicle. (B) KD-BER-LN-428-control with 10 μM **1**. (C) KD-BER-LN-428-LIG3 cells with vehicle. (D) KD-BER-LN-428-LIG3 with 10 μM **1.**
(TIF)
