# Supplementary figures and images for: Interference with DNA repair after ionizing radiation by a pyrrole-imidazole polyamide
Source: PLoS One. 2018 May 1;13(5):e0196803. doi: 10.1371/journal.pone.0196803 (PMC5929528; doi:10.1371/journal.pone.0196803)

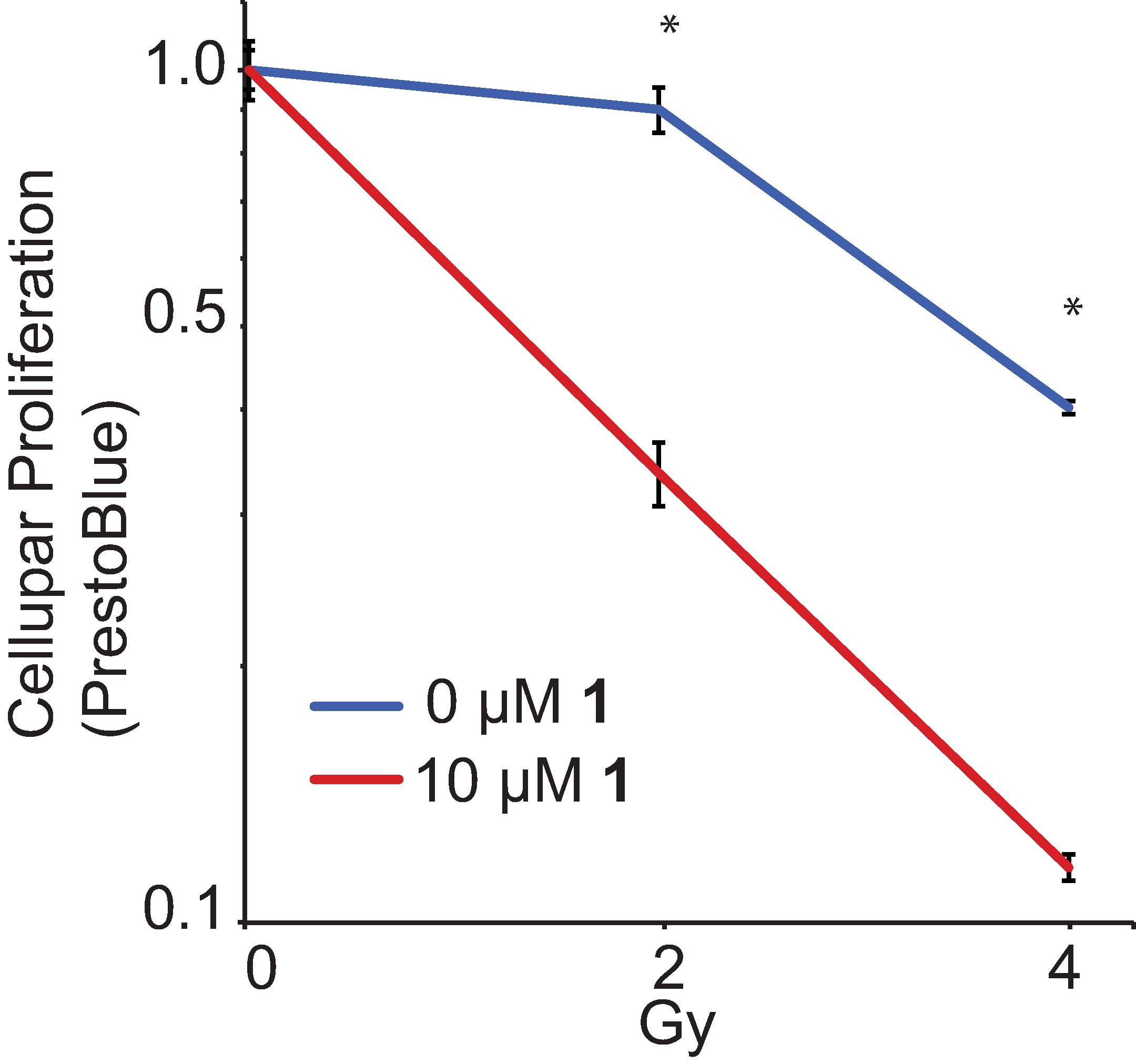

Supplement: S1 Fig — After irradiation, cells were washed twice and fresh media replaced without 1, re-plated at 4000 and 8000 cells/mL in 96 well plates and grown for 14 days. Proliferation was assessed by PrestoBlue assay. * p < 0.01. (TIF) [file pone.0196803.s001.tif]

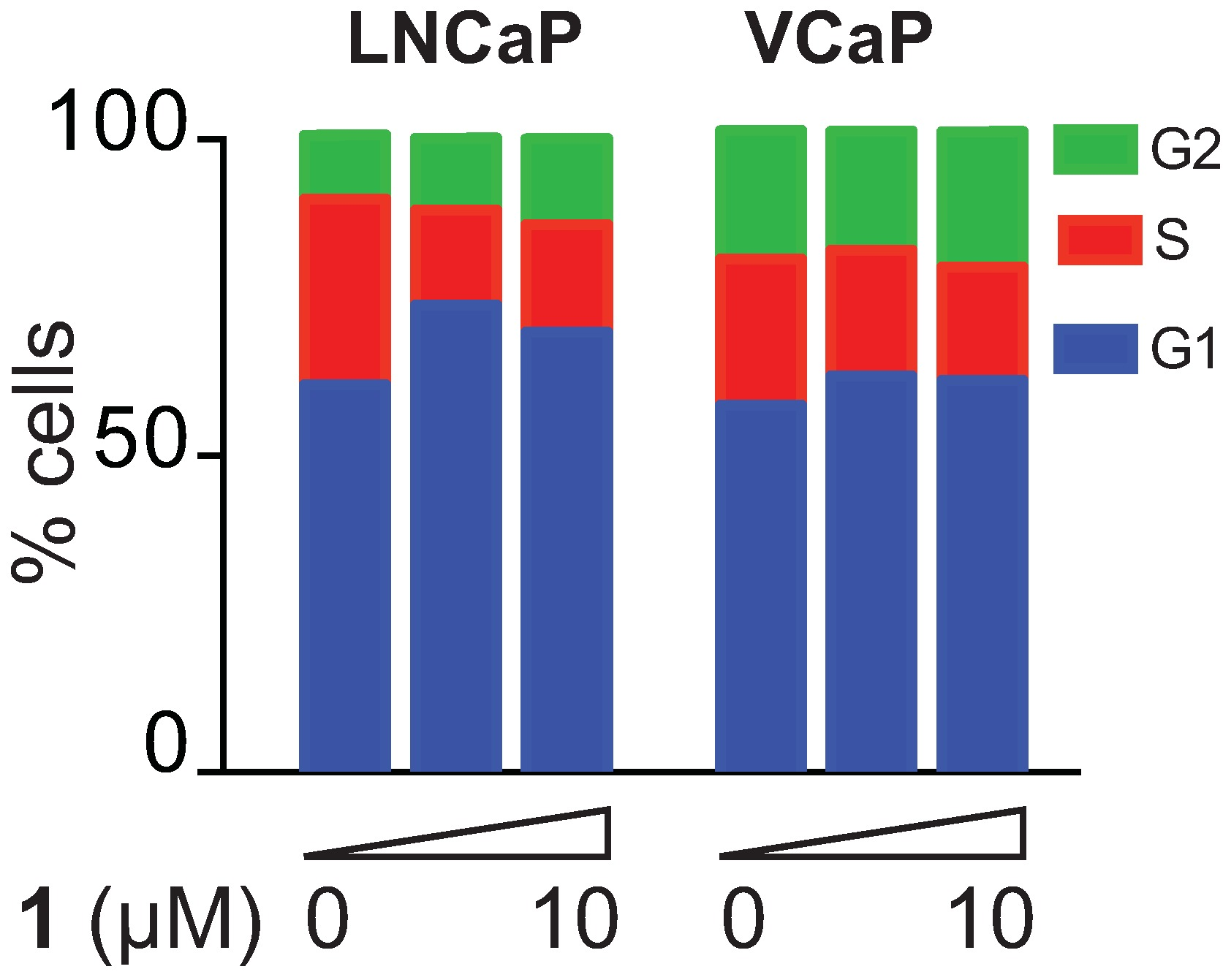

Supplement: S2 Fig — LNCaP and VCaP cells were grown in the presence of polyamide 1 at 5 and 10 μM or vehicle for 48 hours. 1 did not affect the relative distribution of cells in G1, S, or G2 phase in VCaP cells. In LNCaP cells, we observed a small decrease in S phase and small increase in G1, which would not be expected to contribute to increased radiosensitivity. (TIF) [file pone.0196803.s002.tif]

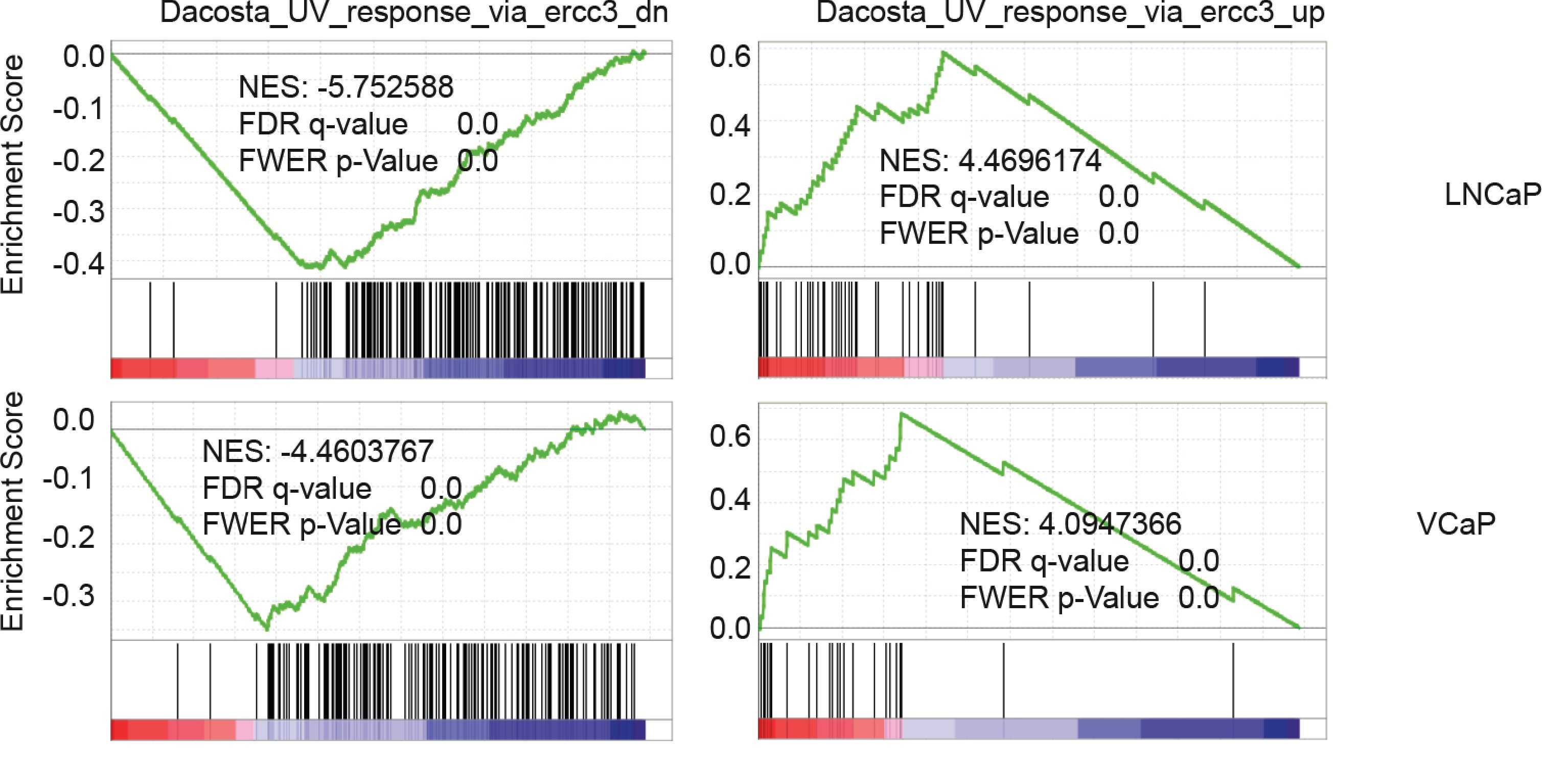

Supplement: S3 Fig — Dacosta_UV_response_via_ercc3_up was the gene set most positively enriched in LNCaP with an enrichment score of 4.47. This set was also enriched in VCaP cells (enrichment score of 4.09). DAcosta_UV_response_via_ercc3_dn was the most negatively enriched in both cell lines. (TIF) [file pone.0196803.s003.tif]

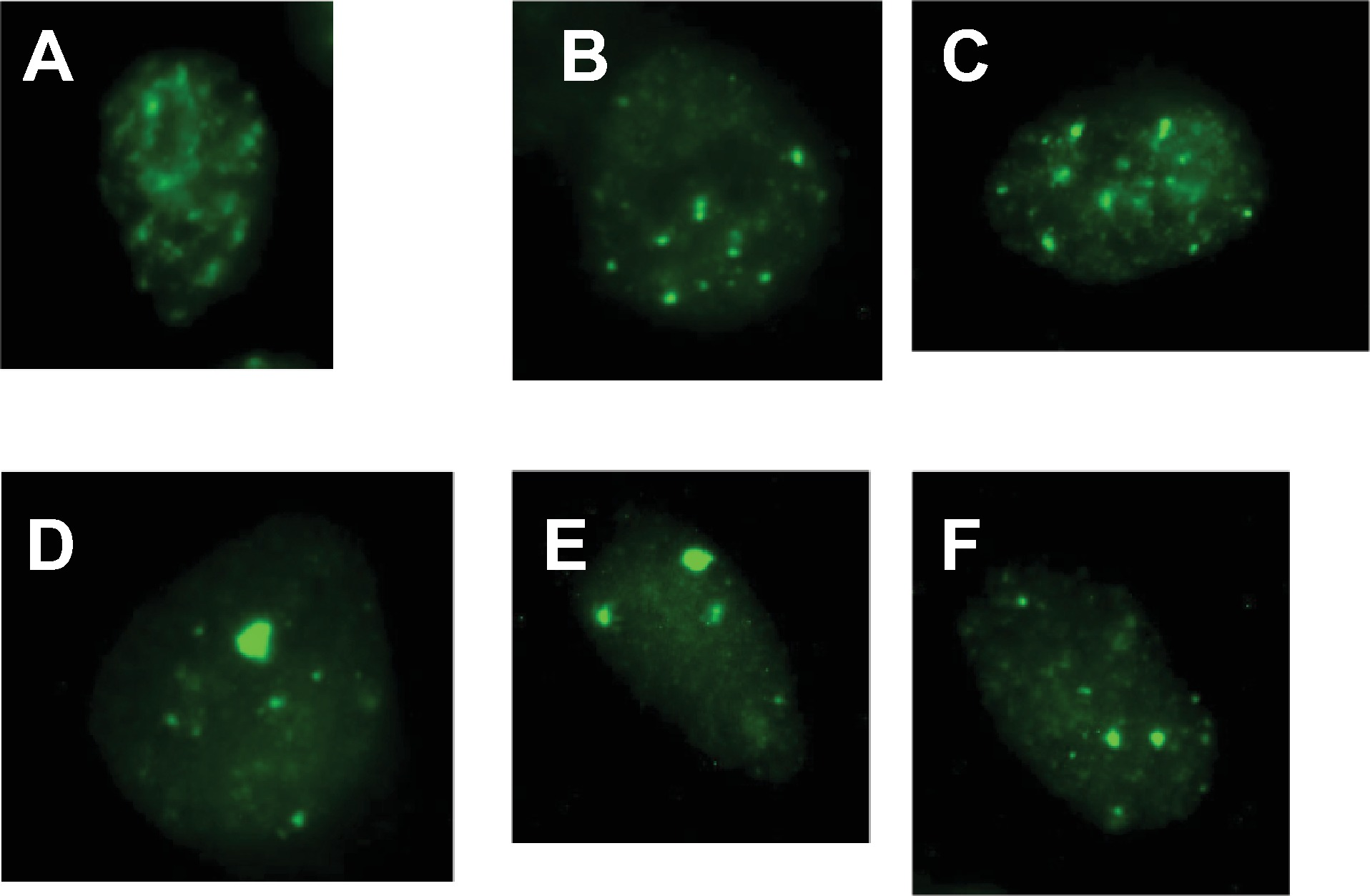

Supplement: S4 Fig — LNCaP and VCaP cells were grown in the presence of polyamide 1 at 5 and 10 μM or vehicle for 24 hours and evaluated by immunostaining for phosphorylated γ-H2AX. (A) LNCaP vehicle. (B) LNCaP 5 μM 1. (C) LNCaP 10 μM 1. (D) VCaP 0 μM 1. (E) VCaP 5 μM 1. (F) VcAP 10 μM 1. (TIF) [file pone.0196803.s004.tif]

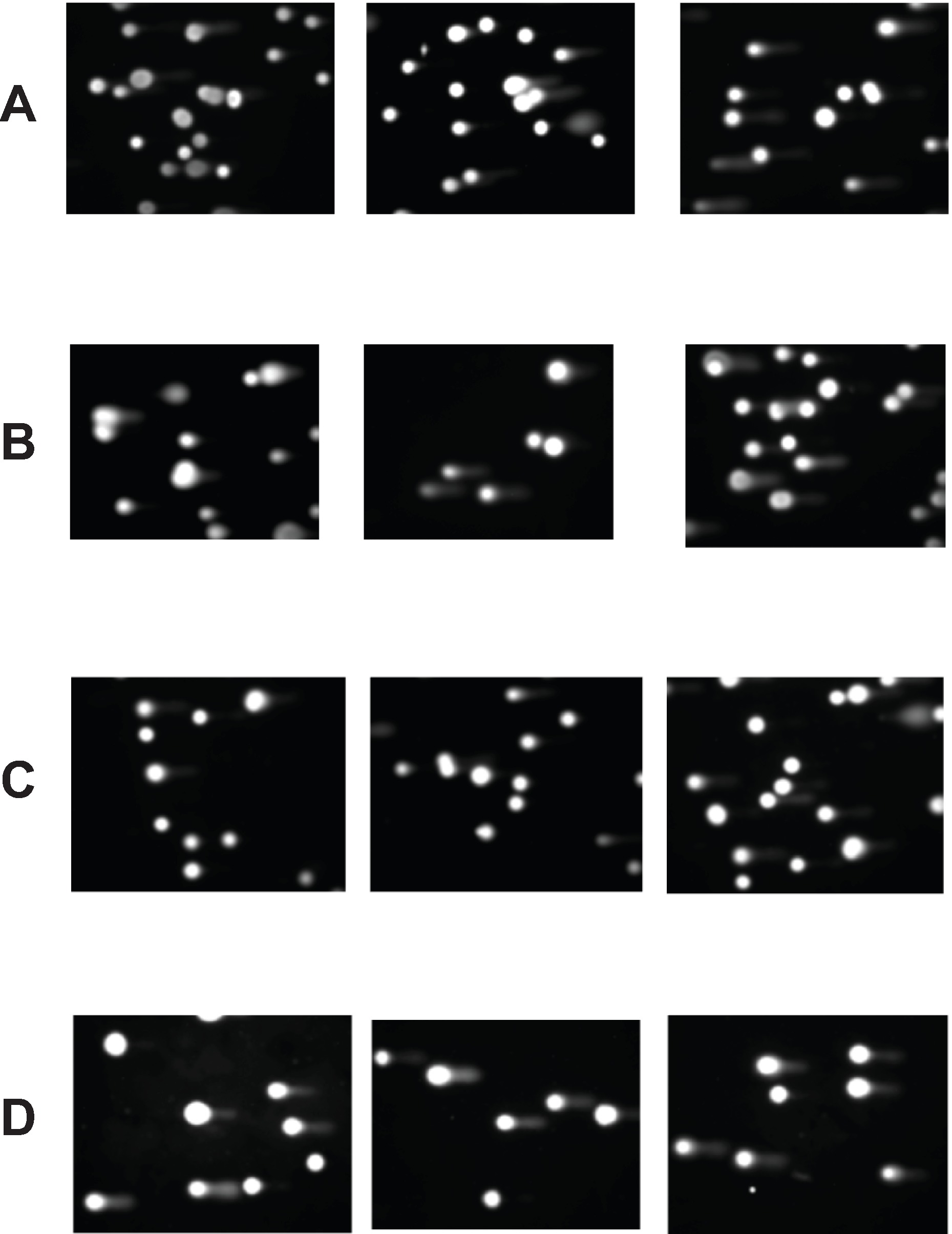

Supplement: S5 Fig — KD-BER-LN-428-LIG3 and KD-BER-LN-428-control cells after treatment with 1 for 24 hours were evaluated by comet assay as described in the manuscript. (A) KD-BER-LN-428-control cells with vehicle. (B) KD-BER-LN-428-control with 10 μM 1. (C) KD-BER-LN-428-LIG3 cells with vehicle. (D) KD-BER-LN-428-LIG3 with 10 μM 1. (TIF) [file pone.0196803.s005.tif]
